# Hydrochar from Spent Coffee Grounds: Characterization and Application for Phenol Removal from Aqueous Solutions Under Static and Dynamic Conditions

**DOI:** 10.3390/toxics14100926

**Published:** 2026-10-19

**Authors:** Sihem Dadi, Hanen Nouri, Beatriz Ledesma, Silvia Román, Salah Jellali, Mejdi Jeguirim, Monia Guiza

**Affiliations:** 1 Laboratory of Process Engineering and Industrial Systems (LRGPSI), National School of Engineers of Gabes, University of Gabes, Gabès 6029, Tunisia; sihem.dadi@enig.rnu.tn (S.D.); hanen.nouri@enigb.tn (H.N.); monia.guiza@enig.rnu.tn (M.G.); 2 Applied Physics Department, Industrial Engineering School, University of Extremadura, Avda. Elvas, s/n, 06006 Badajoz, Spain; beatrizlc@unex.es (B.L.); sroman@unex.es (S.R.); 3 Centre for Environmental Studies and Research, Sultan Qaboos University, Al-Khoud, Muscat 123, Oman; 4 The Institute of Materials Science of Mulhouse (IS2M), University of Haute Alsace, CNRS, UMR 7361, F-68100 Mulhouse, France; mejdi.jeguirim@uha.fr; 5 University of Strasbourg, F-67081 Strasbourg, France

**Keywords:** coffee wastes, hydrothermal carbonization, activation, organics removal, column, reusability

## Abstract

In this work, spent coffee grounds (SCGs) waste is converted into an activated hydrochar (AHC) through hydrothermal carbonization (HTC) at 180 °C followed by steam activation at 850 °C for 1 h. The AHC was firstly characterized and then tested for phenol removal from synthetic aqueous solutions in both batch and column modes. Characterization results show that the AHC is an amorphous material rich in numerous functional groups and has interesting textural properties, with a surface area and a total pore volume of 219.2 m^2^ g^−1^ and 0.156 cm^3^ g^−1^, respectively. These promising properties permit the AHC to successfully remove phenol from aqueous solutions under wide-batch experimental conditions with an adsorption capacity of 226.4 mg g^−1^. Moreover, this process was found to be spontaneous, endothermic, and favorable, involving physical and chemical mechanisms. Furthermore, even in column mode, the AHC retains a strong ability to remove phenol, with an adsorbed amount of 42.3 mg g^−1^ observed for the lowest flow rate (14 mL min^−1^). The phenol breakthrough curves (BTCs) from the column were best fitted with a Thomas model, indicating that limitations of external and intraparticle mass-transfer of phenol are relatively small. Finally, the phenol-loaded AHC can be efficiently regenerated with hot water for at least two cycles. Indeed, the average adsorption efficiencies in the second cycle were evaluated to be 97.7% and 72.7% of those measured in the first cycle, in batch and column modes, respectively. These promising technical results should be consolidated with a detailed life-cycle assessment study in the future.

## 1. Introduction

Coffee is one of the most widely consumed beverages in modern culture and has become an essential part of millions of people’s everyday lives worldwide. According to the International Coffee Organization, worldwide coffee production in 2024/2025 was evaluated at 10.5 million tons, generating an enormous volume of spent coffee grounds (SCGs) waste [1]. More than 90% of the generated SCGs waste is discharged into landfills [2]. In addition to the costs associated with the disposal of these SCGs, their improper management may induce significant risks to ecosystems during their decomposition in landfills [3]. The thermochemical conversion of SCGs into valuable byproducts through well-established methods, such as torrefaction, pyrolysis, gasification, and hydrothermal carbonization (HTC), has been considered an attractive approach for circular economy and environmental sustainability boosting [4]. Due to the large moisture content of SCGs (≈60%), HTC seems to be one of the most suitable conversion methods [5]. HTC technology has been applied to a variety of wet feedstocks including lignocellulosic food-processing waste, animal manure, and sewage sludge [6]. Through this process, biomass volatile matter is transformed into a gaseous fraction, an organic-rich liquid phase that can be valorized for energetic purposes. The residual solid phase is a carbon-rich material that might constitute a value-added byproduct in several sectors including energy recovery, climate change mitigation, sustainable agriculture, and wastewater treatment [6].

Phenol is a hazardous substance produced by various industrial activities including petrochemical plants, pulp and paper mills, dyes, and pharmaceutical synthesis units [7]. Globally, it is estimated that around 10 million tons of phenols are yearly released into the environment [8]. Depending on the type of industry, wastewater with phenol concentrations exceeding 1000 mg L^−1^ may be generated [7]. The presence of phenol in aquatic environments poses significant risks to both human health and ecosystems due to its proved carcinogenicity, high toxicity, and low biodegradability [9]. Therefore, the effective treatment of phenol-contaminated wastewater is an imperative task to be seriously handled. Numerous techniques have been tested for phenol removal from wastewater. They typically involve biodegradation, filtration with specific membranes, and oxidation with advanced processes [10,11,12]. Yet, the large-scale application of these technologies is commonly hindered by either the high cost, low removal effectiveness, or the generation of more toxic byproducts during the treatment process [10]. Conversely, the use of adsorption technology for phenol removal from liquid effluent offers various advantages such as cost-effectiveness, eco-friendless, and practicality [13,14,15]. In this context, Albadarin and Al-Zawahreh [13] published a recent review paper on the selection of high-performance carbon adsorbents for phenol removal from aqueous solutions. These spanned various carbon-based products including activated carbon, graphene, graphene oxide, multiwalled carbon nanotubes, biochars, and various other carbon-based composites [13,16,17,18]. Hydrochars were not included in this list of tested adsorbents.

In the last decade, numerous raw hydrochars have been synthesized from various feedstocks under different HTC experimental conditions [19]. Even if these hydrochars may be rich in various functional groups (i.e., –OH, C=O, C=C, –CH_2_/CH_3_, C–O, and aromatic –CH) [20,21], they usually exhibit poor textural properties with typical specific surface area lower than 50 m^2^ g^−1^ [22]. The application of pristine hydrochars for phenol removal from aqueous solutions usually results in relatively low adsorption capacities [23]. To enhance the physico-chemical properties of hydrochars, and thus their effectiveness in removing organics from effluents, different studies have focused on their optimal modification through chemical and/or physical methods [24,25]. Physical activation of hydrochars at elevated temperatures with oxidizing gases such as water steam or carbon dioxide is usually preferred owing to its lower cost and eco-friendless. Typically, this operation improves the overall properties of hydrochars and especially their surface area and microporosity. For example, the use of a steam activation process has increased the surface area of hydrochars derived from wine waste and date palm pits from around 49.4 to 728 m^2^ g^−1^ [26] and from 14.9 to more than 857.0 m^2^ g^−1^ [27], respectively. The net improvement of the physically modified hydrochar properties usually enhances the efficiency of organics removal from aqueous solutions. In this context, activated orange-peel-derived hydrochars with air or CO_2_ have shown substantial removal of flurbiprofen (a non-steroidal anti-inflammatory drug) from aqueous solutions, with adsorption capacities of more than 158 and 202 mg g^−1^, respectively [28]. However, as far as we know, there were no previous studies on the application of physically activated hydrochars derived from SCGs for phenol removal from liquid effluents.

Moreover, the majority of previous studies regarding the use of raw or activated hydrochars for organics elimination from effluents were carried out under static conditions (batch mode) using small-size flasks [19]. Even if this kind of assay obtains valuable information on fundamental aspects of the adsorption process (e.g., kinetic, isothermic, and thermodynamic behavior), the need for dynamic experiments (i.e., laboratory columns or reactors) has become a crucial task before the upscaling step [29]. To the best of our knowledge, no studies under dynamic conditions were previously conducted on phenol removal from liquid effluents by either raw-, chemically, or physically SCG-derived hydrochars. Moreover, the regeneration of the phenol-loaded hydrochars is commonly carried out in batch mode and using chemical reagents such as NaOH [30]. This method is not sustainable because of its high cost and potential negative threats to the environment. Substituting chemical reagents with eco-friendly eluents (i.e., hot water) may contribute to expense reduction and environmental sustainability boosting. Moreover, an exploration of the regeneration process in dynamic mode in comparison with results in batch mode is rarely conducted.

Therefore, based on these research gaps, the main aim of the current work is to transform huge amounts of SCGs into a valuable hydrochar that can be used several consecutive times for the treatment of phenol-rich effluents. Specifically, this work has the following sub-objectives: (i) synthesis of a steam-activated hydrochar from SCGs, (ii), deep characterization of this material by combining several analytical devices, (iii) evaluation of phenol removal characteristics under wide experimental conditions in batch and also column modes, and (iv) assessment of the regeneration and reusability of the phenol-loaded activated hydrochar by hot water under both static and dynamic conditions.

## 2. Materials and Methods

### 2.1. Raw Hydrochar Preparation

The SCGs were collected from a local coffee shop in the city of Gabes, in the southern part of Tunisia. The hydrochar preparation was performed in a high-pressure stainless-steel reactor with an internal volume of 300 mL. At the beginning of the HTC process, 25 g of SCGs were put in contact with 200 mL of distilled water inside the reactor (mass ratio of 1:8). Then, the reactor was sealed and maintained under autogenous pressure at a temperature of 180 °C and a residence time of 90 min. Upon completion of the HTC process, the reactor was rapidly cooled to room temperature using a cold-water bath for 15 min. Afterwards, the solid residue (hydrochar) was separated from the liquid mixture by filtration through Whatman filter paper, washed three times with distilled water, and dried at 105 °C for 24 h.

The hydrochar production yield (Y (%)) was evaluated by Equation (1):

(1) $$Y(\%)=\frac{M_{\mathrm{HC}}}{M_{\mathrm{SCGs}}}\;\times 100$$

where the M_HC_ and M_SCGs_ are the dry masses of the hydrochar and the spent coffee grounds feedstock, respectively.

### 2.2. Hydrochar Activation

The above obtained hydrochar (HC) was subjected to steam activation in an automated horizontal furnace. The thermal pre-treatment of the hydrochar was carried out under a continuous nitrogen (N_2_) flow of 30 mL min^−1^ at a heating rate of 10 °C min^−1^ up to 850 °C. At this temperature, the heating system was automatically shifted to steam water (70% vol. H_2_O in N_2_) for a duration of 1 h. Finally, the reactor was gradually cooled under N_2_ flow until ambient temperature. The obtained material corresponds to the steam-activated SCGs-derived hydrochar (AHC). It was kept in a glass container and used as is during the characterization and phenol adsorption steps.

### 2.3. Materials Characterization

#### 2.3.1. Elemental and Proximate Analysis

The elemental composition (C, H, N, and O content) of the raw SCGs, the HC, and the AHC was assessed using an automatic elemental analyzer (CHNS-932, LECO Corporation, St. Joseph, MI, USA) according to the standard specification CEN/TS 15104 (2005). The S content was subsequently determined according to the CEN/TS 15289:2006 standard. Finally, the oxygen content was deduced by Equation (2):

O(%) = 100% − C(%) − H(%) − N(%) − S(%) (2)

Moreover, the proximate analysis of these samples (moisture (M), volatile matter (VM), and ash (A) contents) was carried out according to the technical standards CEN/TS 14774-2, CEN/TS 15148, and CEN/TS 14775, respectively. The fixed carbon (FC) content was deduced as below (Equation (3)):

FC = 100% − M(%) − VM(%) − A(%)(3)

#### 2.3.2. Surface Morphology

The surface morphology of the raw SCGs, the HC, and the AHC was examined by scanning electron microscopy (SEM) using a Hitachi S-4800 field-emission scanning electron microscope (FE-SEM) (Hitachi High-Technologies, Tokyo, Japan). The SEM micrographs were acquired in high-vacuum mode (<6 × 10^−4^ Pa) at an operating voltage of 15 kV, a working distance of 12.3 mm, and a magnification of 400, using a secondary electron detection. Before imaging, the samples were sputter-coated with gold to ensure electrical conductivity and prevent charging effects.

#### 2.3.3. Textural Characterization

The textural properties of the HC and the AHC were determined from nitrogen adsorption–desorption isotherm curves measured at 77 K using a Quadrasorb Evo analyzer (Quantachrome Instruments, Boynton Beach, FL, USA). Before analysis, the samples were outgassed at 250 °C for 12 h to remove adsorbed moisture and volatile impurities. The specific surface area (S_BET_ (m^2^ g^−1^)) was calculated according to the Brunauer–Emmett–Teller (BET) model in the relative pressure range of [P/P_0_] = 1.10^−3^–4.10^−5^. The t-plot method was used to estimate the microporous volume (V_mic_, (cm^3^ g^−1^)) and the external surface area (S_EXT_, (m^2^ g^−1^)). The total pore volume was determined from the amount of nitrogen adsorbed at P/P_0_ = 0.99, while the Barrett–Joyner–Halenda (BJH) method was applied to determine the mesopore size distribution.

#### 2.3.4. Surface Chemistry

The existing functional groups at the surface of the SCG, HC, and AHC samples were characterized by Fourier-transform infrared (FTIR) spectroscopy. A Nicolet iS5 FTIR spectrometer (Thermo Fisher Scientific, Waltham, MA, USA) was used to obtain the related spectra over the wavenumber range of 400–4000 cm^−1^ at a resolution of 4 cm^−1^. Moreover, the pH at the point of zero charge (pHpzc) of the AHC was assessed according to the pH drift method for initial pH values varying between 2 and 12 adjusted by 0.1 M NaOH or 0.1 M HCl standard solutions. The pHpzc value is the intersection point between the bisector and the final pH (pH_f_) vs. initial pH (pH_i_) curve. The pH_f_ and pH_i_ were measured using a Basic20 bench pH-meter (Crison Instruments, Alella, Spain).

### 2.4. Phenol Adsorption Study

Phenol, having a chemical formula of C_6_H_5_OH and a molar mass of 94.11 g mol^−1^, was used as an organic pollutant in this work. It was purchased from Fisher Scientific (Pittsburgh, PA, USA). A phenol stock solution at a concentration of 1000 mg L^−1^ was prepared and used throughout this study for the preparation of solutions at desired concentrations. Moreover, phenol concentrations before and after adsorption were measured using a Shimadzu UV-1700 spectrophotometer (Shimadzu Corporation, Kyoto, Japan) at a wavelength of 270 nm.

#### 2.4.1. Batch Mode

The phenol adsorption by AHC in batch mode was conducted through the agitation at 480 rpm by a magnetic stirrer (IKA RT10, Breisgau, Germany) of a specific mass of AHC in 100 mL of synthetic solution at a desired concentration, initial pH, temperature, and a fixed contact time. Unless otherwise mentioned, the default experimental conditions included a contact time of 3 h, an initial pH of 6.0, a liquid solution temperature of 25 °C, an AHC dosage of 2 g L^−1^, and an initial phenol concentration of 100 mg L^−1^. In the current work, the effectiveness of the AHC in removing phenol from aqueous solutions was evaluated under the following parameter variation: (i) contact time for values between 5 and 240 min; (ii) solution temperature for values of 25 °C, 40 °C, and 60 °C; (iii) AHC dosage for an interval of 1 to 7 g L^−1^; (iv) initial pH ranging from 2 to 12; and (v) initial phenol concentration going from 20 to 940 mg L^−1^.

After a given contact time “t”, the phenol adsorbed amount (q_t_(mg g^−1^)) and the phenol removal efficiency R (%) were quantified by Equations (4) and (5), respectively.

(4) $$q_{t}=\frac{C_{0}-C_{t}}{M}\times V$$

(5) $$R_{t}\left(\%\right)=\frac{C_{0}-C_{t}}{C_{0}}\times 100$$

where M (g) is the AHC mass, C_t_ and C_0_ (mg L^−1^) are the phenol concentration at a time “t” and at the beginning of the experiment, and V is the solution volume in the batch setup (L).

Moreover, the observed kinetic data were fitted to three widely used models, namely the pseudo-first order (PFO), pseudo-second order (PSO), and diffusion models. More details about the model equations are depicted in Table S1 ((Equations (S1)–(S4)) in the Supplementary Material File). In addition, the experimental adsorption isotherm data were also examined using three familiar models, namely the Langmuir, Freundlich, and Dubinin–Radushkevich models (Equations (S5)–(S7) in Table S2). Moreover, the agreement between the observed kinetic and isotherm data and the predicted values by the selected models was assessed using the coefficient of determination (R^2^) and the mean absolute percentage errors (MAPE_Kin_ and MAPEIso) as defined below (Equations (6) and (7)):

(6) $${\mathrm{MAPE}}_{\mathrm{Kin}}=\frac{1}{N}\sum_{1}^{N}\frac{\left|\left(q_{t,\exp}-q_{t,\mathrm{cal}}\right)\right|}{q_{t,\exp}}\times 100$$

(7) $${\mathrm{MAPE}}_{\mathrm{Iso}}=\frac{1}{N}\sum_{1}^{N}\frac{\left|\left(q_{e,\exp}-q_{e,\mathrm{cal}}\right)\right|}{q_{e,\exp}}\times 100$$

where q_t,exp_ and q_e,exp_ represent the experimentally measured quantity of phenol adsorbed at time “t” and at equilibrium, respectively, while q_t,cal_ and q_e,cal_ are the corresponding calculated adsorption capacities at time “t” and at equilibrium as determined by the models.

To gain further insight into the phenol adsorption process, the main thermodynamic parameters were determined, including the standard Gibbs free energy change (ΔG°, kJ mol^−1^), enthalpy changes (ΔH°, kJ mol^−1^), and entropy changes (ΔS°, kJ·mol^−1^ K^−1^). They were calculated using the following equations (Equations (8)–(10)) [31]:

ΔG° = −RT × lnKc (8)

ΔG° = ΔH° − T × ΔS°(9)

(10) $$\mathrm{Kc}=\frac{q_{e}}{C_{e}}$$

It is worth mentioning that each batch experiment was performed in triplicate, and the average values are reported in this work.

#### 2.4.2. Column Mode

**Figure 1:**
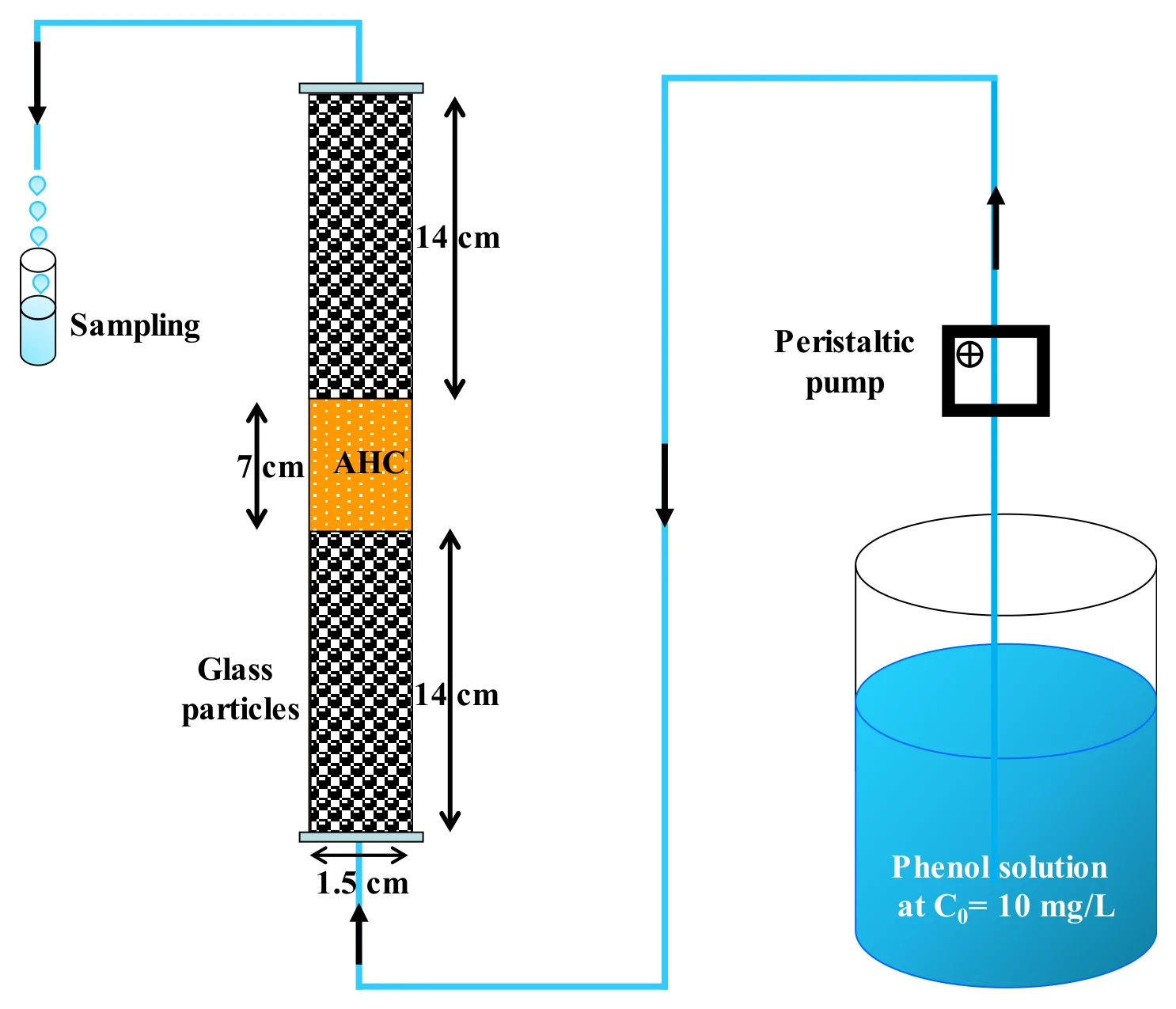
Schematic representation of the column system used for the study of phenol removal by AHC material.

Phenol removal using AHC was also investigated under dynamic conditions using a laboratory glass column with a length of 35 cm and an inner diameter of 1.5 cm (Figure 1). Firstly, the column was packed with 2 g of AHC, corresponding to an approximate height of 7 cm. This material is placed between two thin cellulose fiber meshes and two layers of inert glass particles in order to ensure its stability inside the column. Then, the phenol solution at a constant concentration of 10 mg L^−1^ was injected at the bottom of the column from the feeding tank using a peristaltic pump (EP-1 Econo Pump, Bio-Rad Laboratories, Hercules, CA, USA). This up-flow mode was used in order to avoid any preferential pathways and to ensure a homogenous flow through the AHC material [32]. During this study, the impact of the feed flow rate on the phenol removal efficiency by the ACH was quantified for two values: 14 and 26 mL·min^−1^, which are comparable with those used in previous studies [33,34]. The empty-bed contact times (EBCTs) correspond to the theoretical interaction time between the adsorbent and the dissolvent phenol in the aqueous phase. They were calculated according to the following equation:

(11) $$\mathrm{EBCT}=\frac{V_{b}}{Q}$$

where V_b_ is the packed adsorbent bed volume and Q is the flow rate.

These EBCTs were evaluated at 0.88 and 0.48 min for a flow rate of 14 and 26 mL·min^−1^, respectively. The pressure drop in the column was not experimentally assessed. However, the stability of the flow rate was ensured by the adjustable-flow peristaltic pump and confirmed through regular measurements at the outlet of the column. Moreover, no visible AHC bed expansion was observed during the assays.

The phenol breakthrough curves (BTCs) were established by collecting samples at regular time intervals and analyzing them by UV–visible spectroscopy. The experiments were ended when the relative phenol concentrations (C/C_0_) attained 1. At this stage, it is assumed that the phenol molecules can no longer be removed by the AHC material.

These assays were performed at 25 °C and at the unadjusted natural pH of the feeding solution. All column assays were conducted in duplicate, and the average values are reported in this work.

The experimental BTCs of the phenol pollutant were compared with those obtained for a conservative tracer, NaCl (2 g L^−1^), that does not interact with the AHC particles. The phenol removed amount in the column mode (q_col_) was calculated by the trapezoidal integration method [29] according to Equation (S9) in Table S3. Moreover, the experimental phenol BTCs were fitted with three widely used mathematical models: Bohart–Adams, Thomas, and Yoon–Nelson (Equations (S12)–(S14) in Table S4). More details about these models are given in previous studies [29,35]. As for the batch mode, the agreement between the observed and predicted phenol BTCs was evaluated using the coefficient of determination (R^2^) and the mean absolute percentage error (MAPE_col_) as follows (Equation (12)):

(12) $${\mathrm{MAPE}}_{\mathrm{col}}\;=\;\frac{\sum_{1}^{N}\left|\frac{{\left(\frac{C_{t,\mathrm{Phen}}}{C_{0,\mathrm{Phen}}}\right)}_{\exp}\;-{\left(\frac{C_{t,\mathrm{Phen}}}{C_{0,\mathrm{Phen}}}\right)}_{\mathrm{cal}}}{{\left(\frac{C_{t,\mathrm{Phen}}}{C_{0,\mathrm{Phen}}}\right)}_{\exp}}\right|}{N}\;\times \;100$$

where (C_t,Phen_/C_0,Phen_)_exp_ and (C_t,Phen_/C_0,Phen_)_cal_ correspond to the measured and calculated relative concentrations, respectively. N represents the total number of experimental data.

### 2.5. Adsorbent Regeneration

The regeneration of the AHC was evaluated in both batch and column modes. In batch mode, the regeneration procedure consisted of stirring 2 g of phenol-loaded AHC at 480 rpm in 200 mL of hot distilled water solution (80 °C) for 2 h. The regeneration process was repeated over five successive adsorption–desorption cycles. Similarly, in column mode, the regeneration of the AHC was evaluated using hot distilled water at 80 °C for three consecutive adsorption–desorption cycles and a constant flow rate of 26 mL·min^−1^. For a given cycle, the desorbed mass in column mode was calculated using Equation (S11) in Table S3.

Either in batch or column mode, the regeneration efficiency (RE) after a given adsorption–desorption cycle was calculated using the following equation (Equation (13)):

(13) $$R_{E}\left(\%\right)=\frac{q_{n}}{q_{1}}\times 100$$

where q_1_ and q_n_ (mg·g^−1^) are the adsorbed amount at the first and after the n^th^ regeneration cycle, respectively.

### 2.6. Statistical Analysis

Graphical representations and regression analyses of both calculated and experimental data were performed using Microsoft Excel 2016 (Microsoft Corporation, Redmond, WA, USA) and OriginPro 9.0 (OriginLab Corporation, Northampton, MA, USA). The error bars displayed on the graphs indicate the variability of results obtained from triplicate and duplicate measurements of the batch and column experiments, respectively.

## 3. Results and Discussion

### 3.1. Materials Characterization

Using Equation (1), the production yield of the SCGs-derived hydrochar was evaluated at 57.6%. This relatively high value reflects the non-severity of the HTC conditions (temperature and feedstock-to-water ratio). This value is consistent with those reported in previous studies on SCGs’ HTC conversion under moderate conditions (50–65%) [36]. The steam activation of the HC at 850 °C for 1 h decreases this solid yield to 22.3%, which is equivalent to a burn-off of the hydrochar mass of around 61.3%. This substantial mass loss results from steam–carbon gasification reactions that usually enhance surface area and microporosity development. Comparable production yields (20–35%) have been reported for steam-activated hydrochars from date palm waste at 800–900 °C [27].

#### 3.1.1. Elemental and Proximate Analysis

The proximate and ultimate analysis results of the SCGs, its derived HC, and AHC are summarized in Table 1. The proximate analysis shows clear changes after HTC and also the activation processes. Indeed, the raw feedstock exhibits a high volatile matter content (81.1%), moderate fixed carbon (17.1%), and a low ash content (1.8%). As expected, after HTC, the volatile matter and ash contents have decreased, by around 40.9% and 0.8%, respectively. Conversely, the fixed carbon content has increased by 58.4% (Table 1). This is due to the thermal decomposition of hemicellulose and part of the cellulose components during the HTC process, which promotes dehydration, decarboxylation, and condensation reactions. These reactions remove volatile oxygenated compounds and enrich the solid byproduct in carbon, leading to a higher fixed carbon content [24]. A comparable trend was reported by Cervera-Mata et al. [37] during the HTC of SCGs collected from a university cafeteria in Spain. The steam activation has accentuated the decrease of volatile matter content, reaching a value of only 5.1%. At the same time, the fixed carbon and ash contents have significantly increased to 85.0% and 4.1%, respectively (Table 1). This evolution is associated with an intensified volatilization and the removal of oxygenated functional groups, which together promote carbon enrichment, the development of a more condensed aromatic carbon framework, and the relative concentration of thermally stable mineral phases in the solid byproduct [28].

Regarding the ultimate analysis, as anticipated, results indicate that the AHC exhibits the highest carbon content (92.2 wt.%), which is significantly higher than that of the raw SCGs (57.59 wt.%) and the HC (71.7 wt.%) (Table 1). This highlights the severe carbonization of the AHC due to steam activation and suggests its potential suitability as a high-value carbon material for environmental applications. The combination of HTC with steam activation enriched the carbon content by selectively removing volatile heteroatoms and also oxygenic compounds [26]. Consequently, the oxygen content has decreased from 41.2 wt.% in the raw feedstock to only 7.0 wt.% in the AHC (Table 1). At the same time, this operation has decreased the hydrogen content by around 42% (Table 1). Furthermore, the H/C and O/C are important parameters offering insights into the degree of carbonization, aromatic structural maturity, and stability of hydrochars [25]. Usually, the decrease of H/C and O/C ratios reflects the level of deoxygenation resulting from decarboxylation and dehydration reactions during the HTC and steam activation processes. These reactions typically lead to the removal of oxygen and hydrogen in the form of CO_2_ and H_2_O [25,38]. As a result, the HTC of the SCGs has lowered the atomic ratios of H/C and O/C from 0.019 to 0.001 and from 0.583 to 0.293, respectively. This finding suggests that deoxygenation of SCGs during HTC proceeds mainly through dehydration and decarboxylation, leading to the reduction of oxygen-containing groups and carbon enrichment of the solid [19]. These reactions cause the loss of hydroxyl, carboxyl, and carbonyl functionalities, an increase in aromaticity, and the cleavage of aliphatic side chains, in agreement with previous studies on biomass HTC [6,39]. In addition, as expected, the activation process has significantly decreased the O/C content to 0.057 (Table 1). During steam activation, hydrochar is partially gasified, resulting in the preferential removal of amorphous carbon, residual tar, and volatile matter. In parallel, the cleavage of aliphatic side chains and the elimination of thermally unstable oxygen-containing functional groups contribute to the development of a more condensed aromatic carbon structure and to the enhancement of the material’s textural properties [21,27]. Previous studies have reported a similar trend in H/C and O/C ratio evolution upon HTC and activation [28,39]. For instance, Mena et al. [39] converted *Tectona grandis* seeds into hydrochars via HTC, resulting in a significant decrease in the H/C atomic ratio from 1.584 to 1.045 and the O/C atomic ratio from 0.612 to 0.407. Similarly, this same study showed that hydrochar activation with CO_2_ at 850 °C increased the carbon content from 66.2% to more than 83%. Therefore, the proximate and ultimate analysis suggest that the carbon-rich AHC derived from SCGs waste may have important potential for the effective removal of organics from liquid effluents.

#### 3.1.2. Surface Morphology

**Figure 2:**
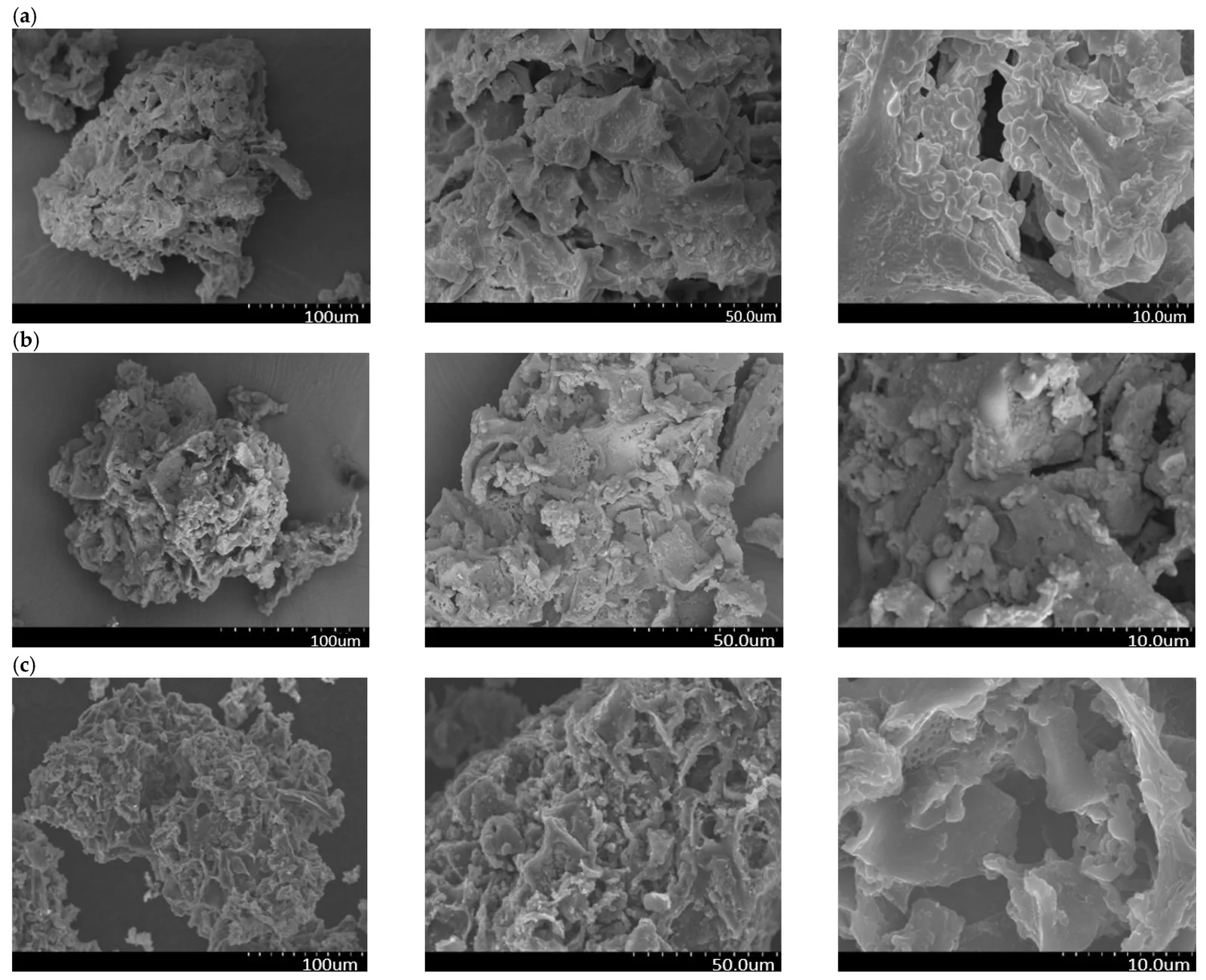
SEM images of (**a**) pristine SCGs, (**b**) SCGs-derived hydrochar, and (**c**) activated-SCGs-derived hydrochar.

SEM micrographs reveal significant morphological differences following the HTC and combined HTC/steam activation processes (Figure 2). The raw feedstock (Figure 2a) exhibits a dense and compact structure with limited visible porosity. After the feedstock HTC, the HC surface became rougher and more fragmented, with the emergence of small pores and cavities (Figure 2b). This suggests a partial degradation of the contained organic matter and the onset of pore formation [27]. The combination of HTC with steam activation processes reveals the formation of a highly porous and interconnected structure, with a well-developed network of macropores and mesopores (Figure 2c). This result is in agreement with that obtained for an activated food-waste-derived hydrochar [40]. The dense pore network observed in the ACH may result in a greater exposed surface area for phenol removal.

#### 3.1.3. Textural Properties

**Figure 3:**
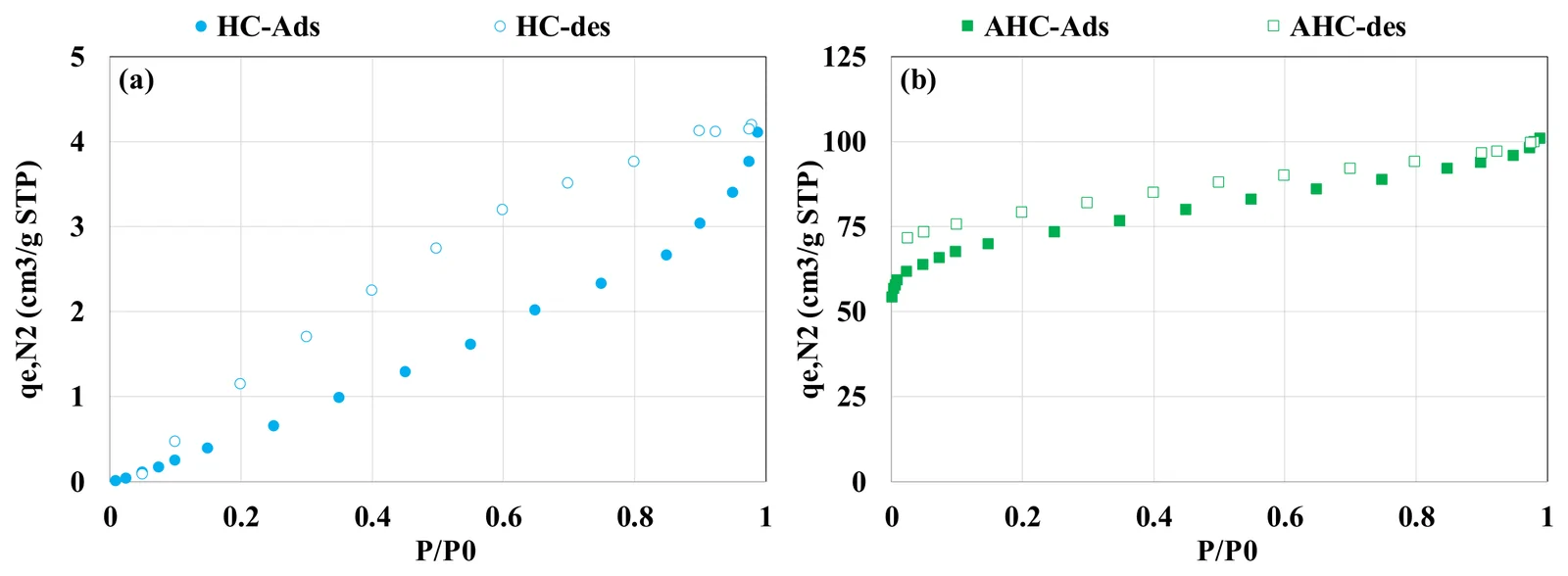
N_2_ adsorption–desorption isotherms of the HC (**a**), and the AHC (**b**) derived from SCGs.

The N_2_ adsorption–desorption isotherm of the HC material displays a sigmoidal shape with limited adsorption and a small hysteresis (Figure 3a), indicating a poorly developed porous structure. In contrast, the AHC-related isotherm (Figure 3b) shows a much greater N_2_ uptake and also a more pronounced hysteresis. This is characteristic of the Type IV isotherm for mesoporous materials [41].

The textural properties of the HC and AHC materials are summarized in Table 2. It clearly shows that the HC material has relatively poor textural properties, which confirms that HTC alone does not generate an extensive pore network. This may be attributed to pore blockage by the residual volatile matter that was unable to migrate to the liquid phase during HTC, and also to the re-polymerized or condensed organic matter [21]. The steam activation produced an activated material with a relatively large specific surface area (219.2 m^2^ g^−1^) and total pore volume (0.156 cm^3^ g^−1^) (Table 2). These values are around 20 and 26 times greater, respectively, than those measured for the HC. Moreover, the AHC’s internal surface and microporosity are relatively important and represent around 89% and 73% of the overall surface area and total pore volume, respectively (Table 2). This result confirms the SEM findings and demonstrates that steam activation has significantly contributed to the development of intraparticle porosity (mesoporosity and microporosity). It is also in agreement with those reported in previous works [27,42]. For instance, Bedoui et al. [27] reported that steam activation of a date-palm-pits-derived hydrochar increased its surface area and microporosity from 14.9 to 857.1 m^2^ g^−1^ and from 0.001 to 0.114 cm^3^ g^−1^, respectively. This improvement indicates the effective development of porosity through pore opening and widening during the activation process. It is worth mentioning that for both HC and AHC materials, the average pore sizes (APS) are higher than the kinetic diameter of phenol molecules (~0.6 nm), thereby allowing their possible internal diffusion within the pore network [43]. The enhanced textural properties of the AHC indicate that this material can effectively remove organics from aqueous solutions.

#### 3.1.4. Surface Chemistry Properties

**Figure 4:**
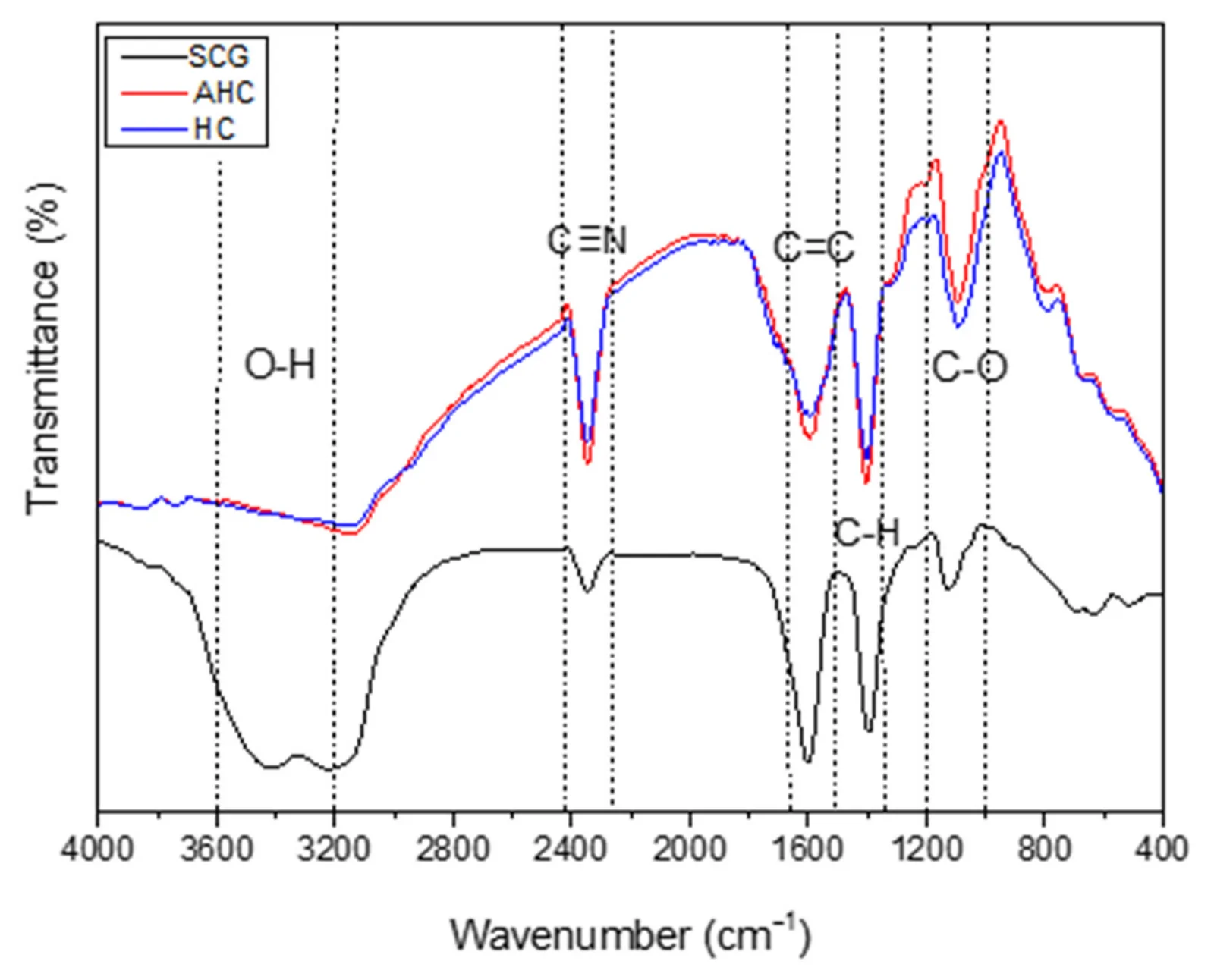
FTIR spectra of SCGs, SCGs-derived hydrochar, and active hydrochar.

The raw SCG, HC, and AHC spectra are depicted in Figure 4. The peak in the region of 3600–3200 cm^−1^ represents the O–H bond stretching in the hemicellulose, cellulose, and lignin components [21]. This peak has significantly decreased after HTC or HTC followed by the activation process. This is attributed to its decomposition by subcritical water with the increase of the HTC temperature. The band (C≡N) observed at around 2350 cm^−1^ is most likely attributable to residual atmospheric CO_2_ and cannot be considered as a characteristic functional group of the investigated materials. Moreover, the bands between 1500 and 1650 cm^−1^ are assigned to the C=C stretching [38], whereas the bands near 1350–1500 cm^−1^ are assigned to C–H bending vibrations and are mainly related to CH_2_ and CH_3_ deformation [44]. These bands remain largely unchanged after HTC or the HTC/activation processes. Furthermore, the bands between 1000 and 1200 cm^−1^ are related to C–O stretching vibrations of hemicellulose, cellulose, and lignin [38]. They are also detected for the three materials. Notably, the HC and AHC spectra largely overlap across the entire wavenumber range, indicating that the surface chemical functionality of the HC is largely preserved after steam activation. This results suggests that the steam activation has primarily modified the structural (see Figure 2) and textural properties (porosity, surface area) (see Figure 3 and Table 2) rather than introducing new surface functional groups.

Moreover, the pHpzc values of the raw SCGs, the HC, and AHC were evaluated at 7.4, 7.1, and 7.8, respectively. The slight decrease after the HTC process suggests that oxygen-containing acidic functional groups were still present on the hydrochar surface after HTC. This finding is in agreement with the FTIR results (See Figure 4). The pHpzc of the AHC was assessed at 7.8. This alkaline pH is due to the thermal degradation of some acidic functional groups (i.e., hydroxyl and carboxyl groups) during the high-temperature steam activation process. Therefore, for aqueous solutions with pH lower than 7.8, the net charge of the AHC will be positive and will favor the attraction of anions.

### 3.2. Phenol Removal in Batch Mode

#### 3.2.1. Effect of Contact Time

**Figure 5:**
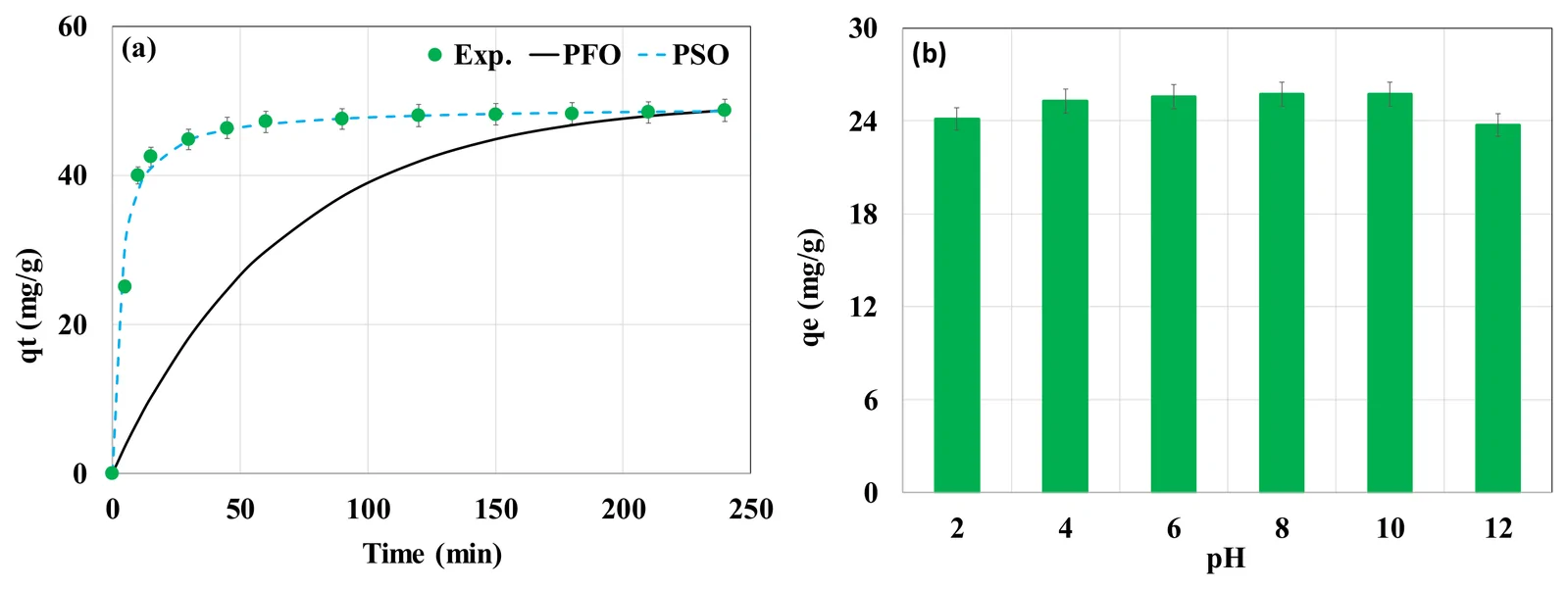
Experimental and modeled kinetic data of phenol removal by AHC material (**a**) (C_0_ = 100 mg L^−1^, pH = 6, D = 2 g L^−1^, T = 25 °C) and effect of initial pH (**b**) (C_0_ = 100 mg L^−1^, t = 3 h, D = 2 g L^−1^, T = 25 °C).

Phenol removal by AHC is a highly time-sensitive process (Figure 5). Indeed, at the beginning of the experiment, the removed phenol amount significantly increases with time owing to the presence of a large number of available active sites on the surface of the AHC. After around 40 min, the rate of adsorption gradually slows down, and it reaches an equilibrium state at around 90–180 min. At this point, most of the AHC active sites are occupied by phenol molecules. A relatively comparable kinetic trend was reported by previous studies on phenol removal on engineered adsorbents [45]. Interestingly, the reported equilibrium contact time was shorter than those found for the removal of phenol by activated carbons derived from industrial and agricultural wastes [33,46,47]. Shorter contact times imply lower energy expenses in up-scaled large-scale systems. Furthermore, the phenol removal process appears to be more limited by diffusion through the water film layer around the AHC particles, as the corresponding diffusing coefficient (D_f_) is almost 1.7 times lower than the intraparticle diffusion coefficient (D_ip_) (Table 3).

The PSO model best fits the experimental data (Table 3 and Figure 5a). Indeed, compared to the PFO model, it exhibits a higher coefficient of determination (R^2^ = 0.942) and lower MAPE_Kin_ (2.8%). Furthermore, the corresponding phenol adsorbed amount at equilibrium (49.2 mg g^−1^) is very close to the experimental value (48.7 mg g^−1^). This result suggests that phenol removal encompasses surface chemical reactions [48].

#### 3.2.2. Effect of Initial Aqueous pH

The effect of initial aqueous pH values on phenol removal by AHC is depicted in Figure 5b. Results show that the phenol adsorbed amount remains quasi-constant for the studied pH range between 4 and 10, with a mean value of 25.3 mg g^−1^. Increasing the initial pH to 12 has slightly decreased the removed amount by around 5.9% (23.8 mg g^−1^). This trend can be attributed to changes in electrostatic interactions between the AHC surface and phenol species. Indeed, for (pH < pKa ≈ 9.9), the phenol exists predominantly in a neutral form. At pH values below the pHpzc of the AHC (7.8), the adsorbent surface carries a positive charge, promoting hydrogen bonding and π–π interactions with neutral phenol molecules. However, for (pH > pka), a significant fraction of phenol is converted into phenolate anions and will be consequently repulsed by the AHC particles that are negatively charged for pH values above 7.8. A equivalent trend was found by Jamilah et al. [49] when studying phenol removal by a bamboo-activated carbon.

#### 3.2.3. Effect of Temperature–Thermodynamic Parameters

The increase of temperature from 25 °C to 60 °C has increased the phenol adsorbed amount by around 7.9% (Table 4). This behavior may be attributed to the thermal expansion of AHC pores facilitating the diffusion of phenol molecules and enhancing their accessibility to the adsorption sites of AHC [31]. The thermodynamic parameters of phenol removal by AHC material were assessed according to the methodology given in Section 2.4.1. Results (Table 4) show a positive △H° value, indicating that phenol removal is an endothermic process [31]. In addition, this value is below 40 kJ·mol^−1^, suggesting that phenol removal is predominantly physical in nature, encompassing interactions as hydrogen bonding or van der Waals forces [50]. Furthermore, the calculated entropy (△S°) values are positive, signifying an increase in disorder at the adsorbent–solution interface, likely due to the disruption of the hydration shell of phenol and the release of water molecules upon adsorption [31]. The calculated △G° values are negative, indicating a spontaneous adsorption process. These △G° values become more negative as the temperature increases, which implies that the phenol adsorption process becomes more spontaneous at higher temperatures [45].

#### 3.2.4. Effect of Dose

**Figure 6:**
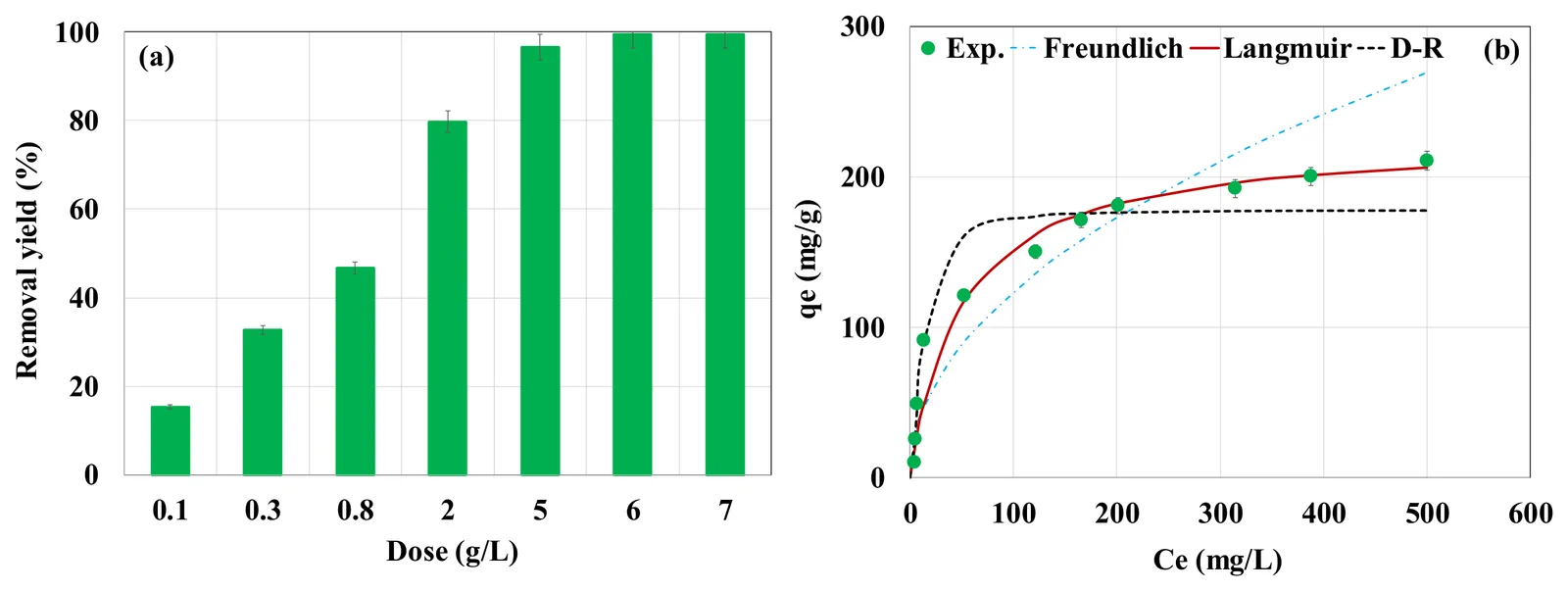
Effect of AHC dosage on phenol removal (**a**) (C_0_ = 100 mg L^−1^, t = 3 h, pH = 6, T = 25 °C) and experimental and theoretical isotherm data vs. initial phenol concentration (**b**) (t = 3 h, pH = 6, D = 2 g L^−1^, T = 25 °C).

The effect of AHC dosage on the efficiency of phenol removal was determined using the experimental conditions described in Section 2.4.1. Results (Figure 6a) indicate that the phenol removal yield increased with increasing AHC dose owing to the existence of a greater number of active adsorption sites. Indeed, the phenol removal yield gradually increases with the rise in AHC dose from 15% at a dose of 0.1 g L^−1^ to more than 99% at a dose of 6 g L^−1^ (Figure 6a). An equivalent trend was observed for phenol removal by hydrochar derived from waste paper sludge [23].

#### 3.2.5. Effect of Initial Phenol Concentration—Isotherm Study

The influence of the initial phenol concentration on its removal by AHC was investigated for a contact time of 3 h, at a non-adjusted pH and an adsorbent dosage of 2 g L^−1^. The related results (Figure 6b) show that the adsorbed phenol amount increased with increasing initial phenol concentration. In fact, this quantity rose from 10.3 mg g^−1^ to more than 210 mg g^−1^ for initial phenol concentrations of 25 and 940 mg L^−1^, respectively. This behavior can be ascribed to the larger concentration gradient between the aqueous phase and the adsorbent surface, which enhances the mass transfer of phenol molecules toward the available adsorption sites [47].

The experimental equilibrium data were fitted with the Langmuir, Freundlich and Dubinin–Radushkevich (D–R) isotherm models, and results are given in Table 5 and Figure 6b. It can be clearly deduced that the Langmuir model best describes the adsorption process, as evidenced by the highest R^2^ (0.963) and lowest MAPE (19.8%) compared to the other models (Table 5) and the closeness of the theoretical curve with the experimental data (Figure 6b). The good agreement of the Langmuir model with the measured data suggests that phenol adsorption onto AHC occurs mainly through monolayer coverage on a homogeneous surface with a finite number of the same adsorption sites [46]. The phenol adsorption capacity was evaluated by this model to be 226.4 mg g^−1^, which is much higher than that of various carbonaceous materials [13]. Furthermore, phenol adsorption by AHC seems favorable as all the calculated values of the Langmuir constant (R_L_ = 1/1 + K_L_ × C_0_) were lower than 1.0. This favorability was also confirmed by the calculated Freundlich parameter “n”, which was largely higher than 1.0 (2.1) (Table 5). Regarding the D–R model, the calculated phenol adsorption capacity was assessed at 178.2 mg g^−1^. This value is more than 15% lower than the experimental one and may be due to the rigorous hypotheses of this model. Furthermore, the mean free energy of adsorption (E) calculated by the D–R model was found to be lower than 8.0 kJ mol^−1^ (Table 5), indicating that the phenol removal process may also include physical mechanisms.

Therefore, based on the results above, phenol adsorption by AHC is an endothermic, spontaneous, favorable process and most probably involves both physical and chemical mechanisms. These mechanisms may encompass electrostatic interactions between phenolic functional groups and oxygenated surface sites, especially under alkaline conditions, complexation with various functional groups, π–π interactions between phenolic rings and aromatic domains of the carbon surface, hydrogen bonding, and pore filling [48].

### 3.3. Phenol Removal in Column Mode

#### 3.3.1. Experimental Results

**Figure 7:**
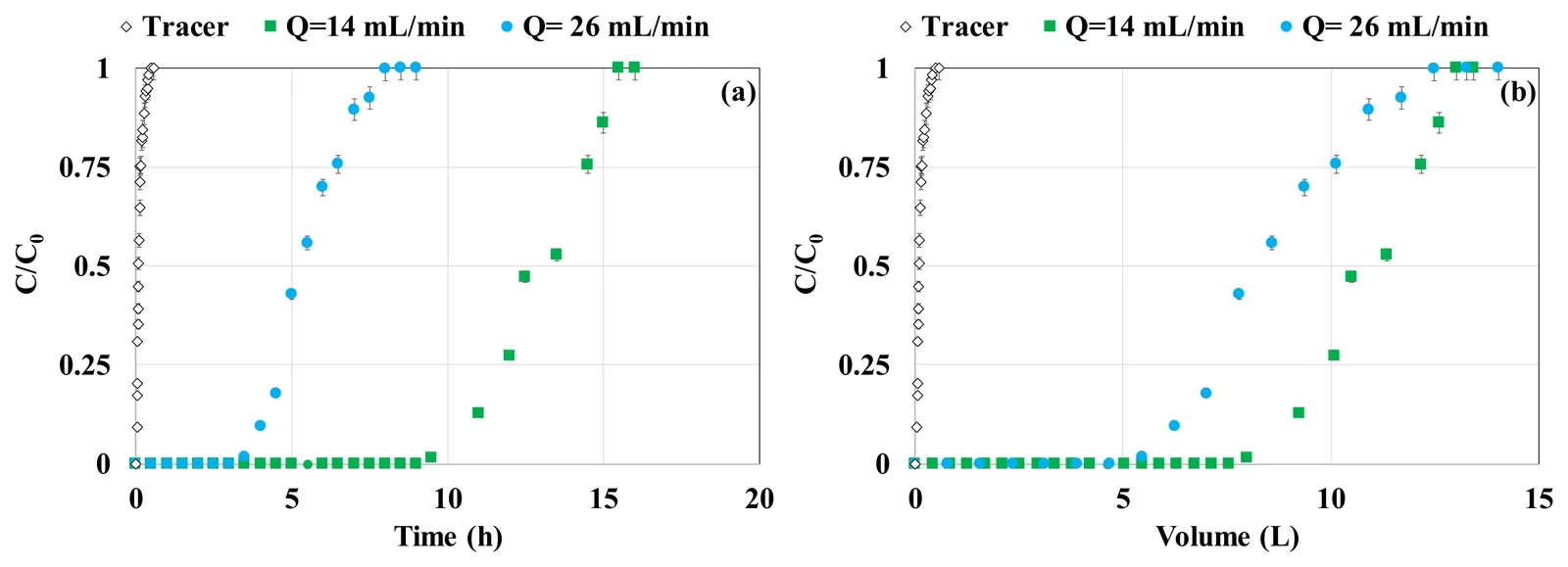
Effect of flow rate on phenol BTCs vs. time (**a**) and vs. injected solution volume (**b**) (C_0_ = 10 mg L^−1^, non-adjusted pH, M = 2 g, T = 25 °C).

Phenol removal by AHC in column mode was investigated for a feeding concentration of 10 mg L^−1^ and an adsorbent mass of 2 g, corresponding to bed height of 7 cm (see Figure 1). Two flow rates were tested: 14 and 26 mL min^−1^. The phenol BTCs were assessed through regular water sample collection and analysis and then compared with that of a conservative tracer (Figure 7).

The BTC of the conservative tracer exhibited a very steep slope. Its breakthrough time (‘t_b_’ when C_t_/C_0_ is equal to 5%) and exhaustion time (‘t_e_’, for C_t_/C_0_ = 95%) were estimated to be only 1.3 min and 22.1 min, respectively. Phenol appears to be effectively removed by AHC, as the “t_b_” and “t_e_” for both applied flow rates were significantly longer than those observed for the conservative tracer (Figure 7a and Table 6). For example, for a flow rate of 14 mL min^−1^, the corresponding “t_b_” and “t_e_” were found to be more than 9.9 and 15.3 h, respectively (Table 6). These values are around 450 and 42 times greater than those of the conservative tracer. Similarly, in term of solution volume, the phenol appears at the outlet of the column after an injected volume of 8.4 and 5.8 L for flow rates of 14 and 26 mL min^−1^, respectively (Table 6 and Figure 7b). These volumes are more than 442 and 305 times higher than that of the conservative tracer, confirming the relatively significant efficiency of phenol removal by AHC.

The phenol adsorption capacities (calculated using Equation (S9)) were assessed to be 42.3 and 36.7 mg g^−1^ for flow rates of 14 and 26 mL·min^−1^, respectively (Table 6). As expected, increasing the flow rate has resulted in a decrease of around 13.2% in the phenol adsorbed mass due to the decrease in contact time between the adsorbate molecules and the active sites of the adsorbent. Indeed, for a flow rate of 14 mL min^−1^, the average residence time (corresponding to a C/C_0_ value of 0.5 of the tracer test) was evaluated to be 5.3 min. This time decreases to only 2.9 min for a flow rate of 26 mL min^−1^. At higher flow rates, the solution passes more rapidly through the column, limiting intraparticle diffusion and mass transfer, which prevents the system from reaching equilibrium and leads to earlier breakthroughs. Conversely, at lower flow rates, the longer contact time enhances diffusion and improves the utilization of adsorption sites, resulting in better removal efficiency [29].

It is worth mentioning that the highest phenol adsorbed mass in column mode (42.3 mg·g^−1^ for 14 mL·min^−1^) is approximately 5.4-fold lower than the Langmuir’s adsorption capacity determined in batch mode (226.4 mg·g^−1^). This finding is primarily due to the shorter contact time between the AHC particles and the phenol molecules inside the column (around 5 min) in comparison with batch mode (3 h). A similar trend was observed for phenol elimination by various activated carbons [33,51,52]. For instance, when shifting from batch to column investigations, Pongsak et al. [51] reported a 2.9-fold reduction in phenol adsorption by corn-cob-derived active carbon.

#### 3.3.2. Mathematical Modeling

**Figure 8:**
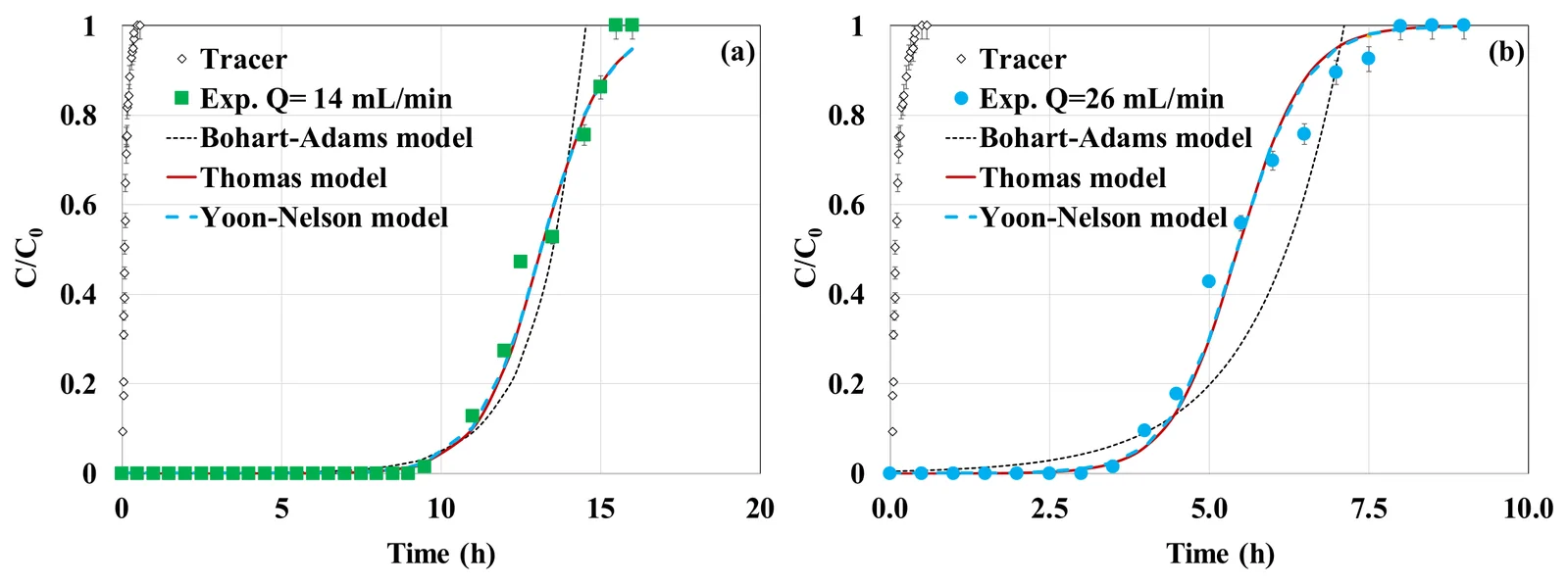
Phenol experimental BTC fitting with Bohart–Adams, Thomas, and Yoon–Nelson models for flow rates of 14 mL min^−1^ (**a**) and 26 mL min^−1^ (**b**) (C_0_ = 10 mg L^−1^, non-adjusted pH, M = 2 g, T = 25 °C).

The phenol BTCs predicted by the Thomas, Bohart–Adams, and Yoon–Nelson models are presented in Figure 8, and the associated parameters are summarized in Table S4. For both tested flow rates, among the three evaluated models, the Thomas model provides the best fitting of the experimental data, as proved by its lower MAPE_col_ and higher R^2^ values (Table S4). This may be attributed to the negligible axial dispersion observed over the tested flow rate range. In addition, the Thomas model assumes that adsorption follows a Langmuir isotherm, which is consistent with the batch results [35,46]. Furthermore, the predicted phenol adsorption capacities by the Thomas model are assessed at 43.2 and 37.1 mg g^−1^ for flow rates of 14 and 26 mL min^−1^, respectively. These values are around 2.1% and 1.1% higher than the experimental ones and confirm that the Thomas model fits the observed data well. The theoretical penetration times (τ_calc_) predicted by the Yoon–Nelson model were also very close to the estimated ones from the experimental data (Table S5), indicating a good agreement between the observed BTCs and the predicted ones by this model. Moreover, the rate constant K_YN_ decreased from 0.0317 to 0.0170 min^−1^ as the flow rate decreased from 26 to 14 mL min^−1^ (Table S5), implying a lower driving force for external mass transfer at lower flow rates [53].

For both flow rates, the Bohart–Adams model did not appropriately restitute the experimental data, as evidenced by the high calculated MAPE_col_ (64.0–95.1%) and the relatively low corresponding R^2^ (0.606–0.861) (Table S5). Moreover, this model largely failed in predicting the last part of the experimental BTCs (Figure 8a,b). A comparable trend was previously reported for phenol and other pollutants by carbonaceous materials [29,35,53].

### 3.4. Phenol Removal Efficiency Comparison with Other Materials

Table 7 presents a comparison of the AHC adsorption capacity in batch and column modes with that of other hydrochars. Given the limited number of studies on hydrochars, this comparison was extended to biochars generated from different biomasses. The phenol adsorption capacity in batch mode (226.4 mg g^−1^) or the removed amount at equilibrium in column mode (42.3 mg g^−1^) is within the range reported for engineered hydrochars and biochars (Table 7). Nevertheless, the direct comparison is very difficult and should be interpreted cautiously, because adsorption efficiency is strongly influenced by the material properties as well as the adsorption experimental conditions, such as the initial aqueous pH, initial phenol concentration, adsorbent mass, contact time, temperature, and the solution chemical composition. These experimental conditions differ considerably among the studies depicted in Table 7.

### 3.5. Adsorbent Regeneration

#### 3.5.1. Batch Mode

**Figure 9:**
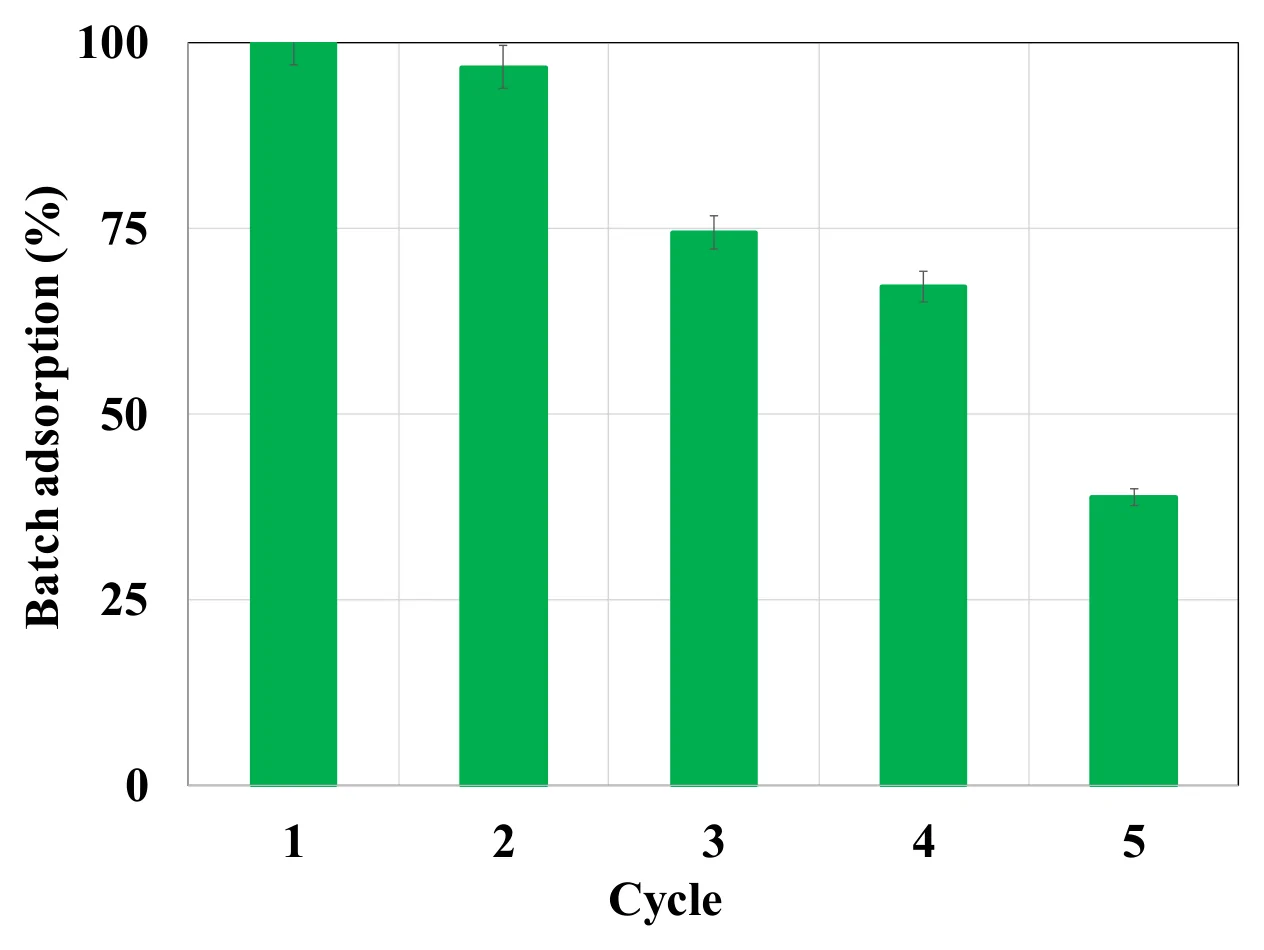
Phenol-loaded AHC regeneration vs. adsorption–desorption cycles in batch mode (hot distilled water at 80 °C, t = 2 h).

The regeneration of the AHC in batch mode was studied according to the experimental conditions given in Section 2.5. Results (Figure 9) show that the phenol adsorption capacity is slightly affected at the second cycle, with a decrease of around 3.3%. Then, this capacity significantly decreases with the adsorption–desorption cycles and reaches 74.4%, 67.2%, and less than 39% at the third, fourth, and fifth cycle, respectively (Figure 9). This decrease may be mainly due to phenol accumulation within the microporous structure, a partial pore blockage, and a reduced availability of active sites [59]. Secondary effects, including minor surface modifications and physical particle abrasion, may further contribute to this efficiency loss. It is important to underline that our AHC exhibits a better regeneration performance in comparison with other engineered hydrochars [13,60]. For instance, for a cellulose-derived hydrochar, Wibiyan et al. [60] showed that phenol adsorption efficiency decreased by approximately 39%, 67%, 92%, and more than 97% after the second, third, fourth, and fifth adsorption–desorption cycles, respectively.

#### 3.5.2. Column Mode

**Figure 10:**
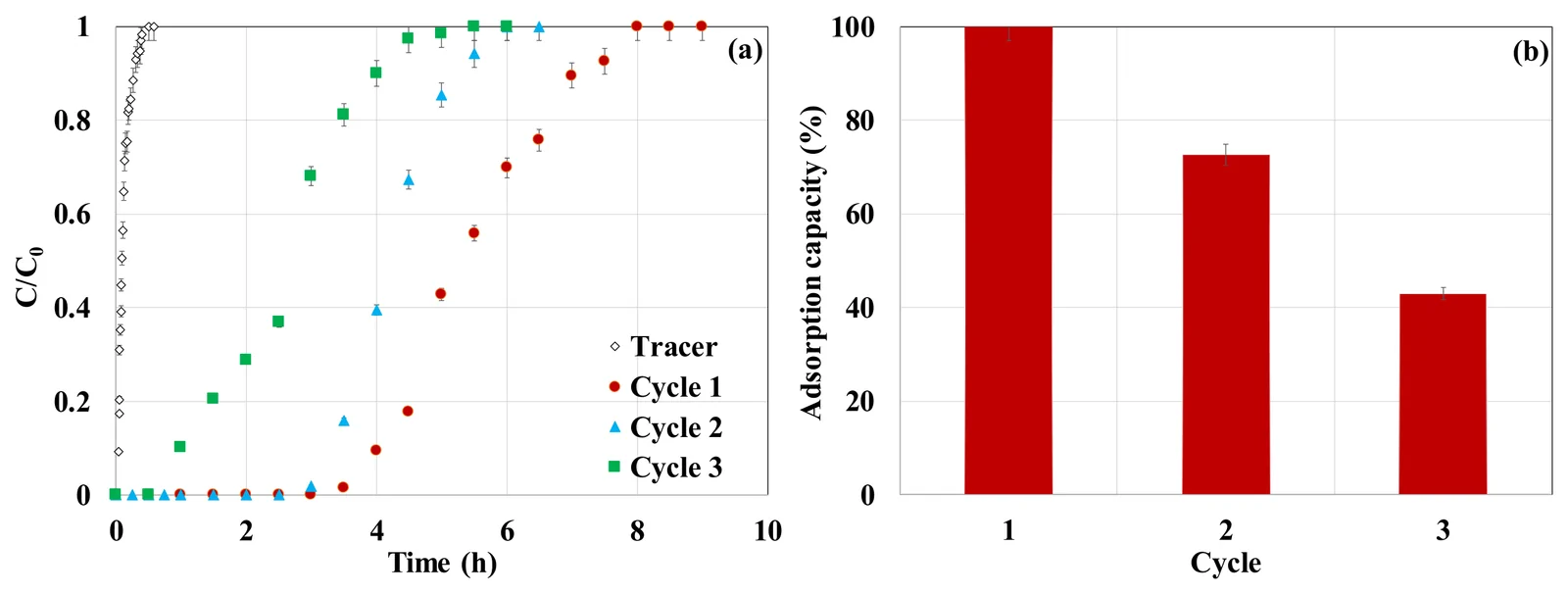
Phenol experimental BTCs (**a**) and adsorption capacity decrease (**b**) vs. adsorption/desorption cycles in column mode (hot distilled water at 80 °C, Q = 26 mL min^−1^).

The phenol desorption efficiency in column mode was conducted with hot water for a flow rate of 26 mL min^−1^ and three successive adsorption–desorption cycles. The related phenol adsorption BTCs are displayed in Figure 10a. It clearly shows an earlier apparition and equilibrium with the increase of regeneration cycle numbers, indicating a net decrease in phenol adsorption efficiency. The phenol adsorbed amounts were evaluated by Equation (S9) as 36.7, 26.7, and 15.8 mg g^−1^ for the regeneration cycles 1, 2, and 3, respectively. In comparison with cycle 1 (100%), the phenol adsorption efficiencies decrease to 72.7% and 42.9% for cycles 2 and 3, respectively (Figure 10b). As in batch mode, the decline in the column’s regeneration efficiency is likely associated with the partial irreversibility of phenol molecules adsorbed on high-energy sites of the adsorbent, such as micropores and specific acidic or aromatic functional groups. These reasons hinder the phenol’s complete desorption during the treatment with hot water. In addition, repeated regeneration cycles in hot water may induce subtle changes in surface chemistry and partial saturation of the adsorption sites by strongly retained or oxidized phenolic species, leading to a progressive loss of accessible active sites for phenol uptake [53]. A similar trend was reported for the regeneration of phenol-loaded olive-pomace-derived material using a 0.1 M HCl solution heated at 60 °C [52].

This regeneration study shows that our AHC can be used at least two consecutive times for phenol removal from aqueous solutions after intermediary desorption steps with hot water. This will offer important economic and environmental benefits in real-case situations. Nevertheless, sustainable management of the desorbing solution remains a key factor for the success of our overall suggested strategy. In this context, this solution may be treated by specific methods such as advanced oxidation processes [61].

SCGs-derived activated hydrochar can be considered a promising material for phenol removal from effluents. However, our suggested approach requires a detailed life-cycle study in order to precisely assess its related negative and positive environmental impacts, its economic viability, and, therefore, its real application potential [62].

## 4. Conclusions

In this study, spent coffee grounds were hydrothermally carbonized at 180 °C for 90 min, and the resulting hydrochar was then activated with steam at 850 °C. This activated hydrochar exhibits interesting structural, textural, and surface chemical properties, in comparison with various engineered chars derived from other biomasses. This activated hydrochar demonstrates significant efficiency of phenol removal from aqueous solutions, with a Langmuir adsorption capacity in batch mode of more than 226 mg g^−1^. Even under dynamic conditions, this activated hydrochar retains a high phenol adsorption ability of around 42 mg g^−1^. The phenol removal was found to be spontaneous and endothermic and involves a combination of physical and chemical mechanisms. The study of the regeneration of activated hydrochar shows that it can be used at least two consecutive times for phenol removal from aqueous solutions. In the future, the validation of these promising results will be carried out with real industrial wastewater. Moreover, the optimal treatment of highly concentrated phenol desorbing solutions will be performed through the use of advanced oxidation processes. A life-cycle assessment study of the whole approach, including all steps related to feedstock collection and pre-treatment and activated hydrochar production and application, is a priority.

## Figures and Tables

**Table 1:** Proximate and ultimate analysis results of the used SCGs and their derived hydrochar and activated hydrochar (VM: volatile matter, FC: fixed carbon, A: ash).

Material	Proximate Analysis			Ultimate Analysis					
	VM (%)	FC (%)	A (%)	C (%)	H (%)	S (%)	O (%)	O/C	H/C
SCG	81.1	17.1	1.80	57.5	1.12	0.19	41.2	0.583	0.019
HC	40.9	58.4	0.77	71.7	0.14	0.16	28.0	0.293	0.001
AHC	5.1	85.0	4.08	92.2	0.64	0.13	7.0	0.057	0.006

**Table 2:** Textural parameters of the SCG-derived hydrochar and activated-SCG-derived hydrochar.

Material	S_BET_ (m^2^ g^−1^)	S_ext_ (m^2^ g^−1^)	TPV (cm^3^ g^−1^)	V_micro_ (cm^3^ g^−1^)	V_meso_ (cm^3^ g^−1^)	APS (nm)
HC	10.9	3.3	0.006	0.001	0.005	2.3
AHC	219.2	24.8	0.156	0.114	0.042	2.8

**Table 3:** Kinetic models’ adsorption parameters for phenol removal by AHC material.

Kinetic Model	Parameter	Value
PFO	q_e,exp_ (mg g^−1^)	48.7
	q_e_ (mg g^−1^)	50.1
	K_1_ (min^−1^)	0.0151
	R^2^	0.606
	MAPE_Kin_ (%)	29.0
PSO	q_e_ (mg g^−1^)	49.2
	K_2_ (g mg^−1^ min^−1^)	0.0067
	R^2^	0.942
	MAPE_Kin_ (%)	2.8
Diffusion	D_f_ (×10^−13^ m^2^ s^−1^)	0.719
	R^2^	0.708
	D_ip_ (×10^−13^ m^2^ s^−1^)	1.195
	R^2^	0.953

**Table 4:** Effect of temperature and thermodynamic parameters of phenol removal by AHC material.

T (K)	q_e_ (mg g^−1^)	ΔG (kJ mol^−1^)	ΔH (kJ mol^−1^)	ΔS(J mol^−1^ K^−1^)
298.15	171.5	−4.048	5.405	0.031
313.15	181.5	−4.411		
333.15	185.0	−5.151		

**Table 5:** Isotherm models’ adsorption parameters for phenol removal by AHC material.

Isotherm	Parameter	Value
Freundlich	n	2.1
	K_F_	13.1
	R^2^	0.898
	MAPE_Iso_ (%)	33.6
Langmuir	K_L_ (L mg^−1^)	0.021
	q_max_ (mg g^−1^)	226.4
	R^2^	0.963
	MAPE_Iso_ (%)	19.8
D–R	q_m_ (mg g^−1^)	178.2
	E (kJ mol^−1^)	2.5
	R^2^	0.922
	MAPE (%)	23.5

**Table 6:** Effect of flow rate on phenol BTC properties and removal efficiency in column mode.

Assay	Breakthrough Time (min)	Exhaustion Time (min)	Breakthrough Volume (L)	Exhaustion Volume (L)	Removed Amount (mg g^−1^)
Tracer, Q = 14 mL min^−1^	1.3	22.1	0.019	0.309	-
Phenol, Q = 14 mL min^−1^	597.7	919.1	8.4	12.9	42.3
Phenol, Q = 26 mL min^−1^	222.9	460.0	5.8	12.0	36.7

**Table 7:** Comparison of phenol removal efficiency by AHC in batch and column modes with that of other hydrochars and/or biochars (T: HTC or pyrolysis temperature; t: residence time: G: heating gradient; C_0_: phenol initial concentration; D: hydrochar dose; ta: contact time for batch adsorption assays; Ta: temperature of the adsorption system; M: material mass; Q: feeding flow rate; Z: bed height; q_e_: phenol adsorbed amount).

Feedstock	Targeted Material	Pre-Treatment Conditions	HTC, Pyrolysis, or Carbonization Conditions	Post-Treatment Conditions	Adsorption Conditions	q_e_ (mg g^−1^)	References
Batch mode							
Paper sludge	Hydrochar	-	HTC at T = 300 °C; t = 15 min; G = 3.42 °C/min	-	C_0_ = 10–150 mg L^−1^ pH = 8; ta = 1400 min; Ta = 25 °C	1.2	[23]
Areca nut waste	Activated biochar	Impregnation with concentrated H_2_SO_4_	Pyrolysis at T = 500 °C; t = 4 h.	Activation with succinic anhydride in acetone solution	C_0_ = 1–20 mg L^−1^; D = 2 g L^−1^; pH = 5; ta = 12 h; Ta = 25 °C	5.4	[46]
Sewage sludge	Pyrolyzed hydrochar	-	HTC at T = 260 °C; t = 6 h	Impregnation with pluronic F127 and HCl solution, then pyrolysis at T = 600 °C and t = 2 h	C_0_ = 24–300 mg L^−1^; pH = 7; D = 4 g L^−1^; ta = 2 h; Ta = 25 °C	22.6	[30]
Banana leaves	Biochar	-	Pyrolysis at T = 400 °C; t = 2 h; G = 10 °C min^−1^	-	C_0_ = 2.5–350 mg L^−1^; D = 1 g L^−1^; pH = 7; ta = 22 h; Ta = 25 °C	16.5	[47]
			Idem. T = 500 °C			34.3	
Coffee Husk	Biochar	-	Pyrolysis at T = 400 °C; t = 2 h; G = 10 °C min^−1^	-	C_0_ = 40 mg L^−1^; D = 1 g L^−1^; pH = 7; ta = 22 h; Ta = 25 °C	27.5	[47]
			Idem. T = 500 °C			43.0	
Corn cobs	Activated carbon	Impregnation with 85% H_3_PO_4_ solution	Carbonization at T = 500 °C; t = 2 h	-	C_0_ = 100–500 mg L^−1^; D = 10 g L^−1^; ta = 6 h; Ta = 25 °C	52.3	[33]
Sugarcane bagasse	Activated carbon	Impregnation with 0.1 M ZnCl_2_ solution	T = 400 °C t = 1 h	-	C_0_ = 150 mg L^−1^; pH = 5.5; D = 2 g L^−1^; ta = 90 min; Ta = 40 °C	64.6	[50]
Crab eye plant (*Abrus precatorius*)	Activated biochar	-	Pyrolysis at T = 750 °C; t = 5 h	Impregnation with a saturated NaOH solution, then pyrolysis at T = 550 °C, t = 2.5 h, G = 5 °C min^−1^	C_0_ = 25–150 mg L^−1^; pH = 8; D = 0.4 g L^−1^; ta = 2 h; Ta = 30 °C	169.4	[54]
Macadamia nut shell	Activated biochar	-	Pyrolysis at T = 950 °C; G = 5 °C mi^n−1^	Activation with CO_2_ at 950 °C for 4 h	C_0_ = 50–300 mg L^−1^; D = 0.1 g L^−1^; pH = 6; ta = 10 h; Ta = 25 °C	588.2	[51]
Spent coffee grounds	Activated hydrochar	-	HTC at T = 180 °C; t = 1.5 h	Activation with water steam at T = 850 °C for 1 h	C0 = 20–940 mg L^−1^; D = 2 g L^−1^; pH = 6; ta = 3 h; Ta = 25 °C	226.4	This study
Column mode							
Coconut shells	Activated carbon	-	Pyrolysis at T = 600 °C; t = 1.5 h	Activation with CO_2_ at 600 °C for 10 min	C_0_ = 20 mg L^−1^; Z = 2 cm; Q = 1 mL min^−1^; Ta = 25 °C	0.06	[55]
Date palm pits	Activated carbon	Impregnation with 54% H_3_PO_4_ solution	Carbonization at T = 547 °C; t = 2.1 h	-	C_0_ = 10 mg L^−1^ Z = 10 cm; Q = 5 mL min^−1^; pH = 7; Ta = 23 °C	0.17	[56]
Areca nut waste	Activated biochar	Impregnation with concentrated H2SO_4_ solution	Pyrolysis at T = 500 °C; t = 4 h.	Activation with succinic anhydride in acetone solution	C_0_ = 7.89 mg L^−1^; pH = 5; M = 1 g; Q = 0.5 mL min^−1^	2.7	[46]
Rice husk	Activated carbon	Impregnation with 45% H_3_PO_4_ solution	Carbonization at T = 575 °C; t = 4 h	-	C_0_ = 100 mg L^−1^; Z = 10 cm; Q = 9 mL min^−1^	14.6	[57]
Rice husk	Activated biochars	Impregnation with ZnCl_2_ solution at a mass ratio of 1:1	Pyrolysis at T = 600 °C; t = 1 h; G = 10 °C min^−1^	-	C_0_ = 400 mg L^−1^; M = 5 g; pH = 5.6; Q = 1 0 mL min^−1^; Ta = 23 °C	17.5	[53]
Corn cobs	Activated carbon	Impregnation with 85% H_3_PO_4_ solution	Carbonization at T = 500 °C; t = 2 h	-	C_0_ = 500 mg L^−1^; M = 12 g; Q = 18.3 mL min^−1^; Ta = 25 °C	32.1	[33]
Sugarcane bagasse	Activated biochar	Impregnation with zinc acetate solution at a mass ratio of 1:1.3	Pyrolysis at T = 800 °C; t = 2 h	Activation with CO_2_ at 800 °C for 1 h	C_0_ = 50 mg L^−1^; Q = 1.5 mL min^−1^; pH = 7	88.2	[58]
Macadamia nut shell	Activated biochar	-	Pyrolysis at T = 950 °C; G = 5 °C min^−1^	Activation with CO_2_ at 950 °C for 4 h	C0 = 150 mg L^−1^; M = 13 g Q = 6 mL min^−1^; Ta = 30 °C	201.6	[51]
Spent coffee grounds	Activated hydrochar	-	HTC at T = 180 °C; t = 1.5 h	Activation with water steam at T = 850 °C; t = 1 h	C_0_ = 10 mg L^−1^; Q = 14 mL min^−1^; M = 2 g; pH = 6; Ta = 25 °C	42.3	This study

## Data Availability

The raw data supporting the conclusions of this article will be made available by the authors on request.

## Supplementary Materials

The following supporting information can be downloaded at: https://www.mdpi.com/article/10.3390/toxics14100926/s1, Table S1: Kinetic models used for the study of phenol removal by SDGs-derived hydrochars; Table S2: Isotherm models used for the study of phenol removal by SDGs-derived hydrochars; Table S3: Equations used for the calculus of phenol adsorbed and desorbed masses in column mode; Table S4: Models used for the fitting of phenol BTCs in column mode; Table S5: Model parameters used for the fitting of the phenol experimental BTCs in column mode.
