# Supplementary material for: Hydrochar from Spent Coffee Grounds: Characterization and Application for Phenol Removal from Aqueous Solutions Under Static and Dynamic Conditions
Source: Toxics. 2026 Oct 19;14(10):926. doi: 10.3390/toxics14100926 (PMC13709396; doi:10.3390/toxics14100926)
Supplement: Supplementary file 1 [file toxics-14-00926-s001.zip › toxics-4566625-supplementary.pdf]

Hydrochar from Spent Coffee Grounds: Characterization and Application for Phenol Removal from Aqueous Solutions Under Static and Dynamic Conditions

Sihem Dadi[1], Hanen Nouri[1], Beatriz Ledesma[2], Silvia Román[2], Salah Jellali[3,*], Mejdi Jeguirim[4], Monia Guiza [1]

[1] Laboratory of Process Engineering and Industrial Systems (LRGPSI), National School of Engineers of Gabes, University of Gabes,6029, Tunisia; sihem.dadi@enig.rnu.tn (S.D.); hanen.nouri@enigb.tn (H.N.); monia.guiza@enig.rnu.tn (M.G.).
[2] Applied Physics Department, Industrial Engineering School, University of Extremadura, Avda. Elvas, s/n, 06006 Badajoz, Spain; beatrizlc@unex.es (B.L.); sroman@unex.es (S.R.).
[3] Centre for Environmental Studies and Research, Sultan Qaboos University, Muscat, Al-Khoud 123, Oman; s.jelali@squ.edu.om (S.J.)
[4] The Institute of Materials Science of Mulhouse (IS2M), University of Haute Alsace, University of Strasbourg, CNRS, UMR 7361, F-68100 Mulhouse, France; mejdi.jeguirim@uha.fr (M.J.)
*: Correspondence

Supplementary materials

Table S1. Kinetic models used for the study of phenol recovery by SDGs-derived hydrochars (q_t and q_e (mg·g^{-1}) denote the adsorption capacities at time t and at equilibrium, respectively, K_1 (min^{-1}) and K_2 (g·mg^{-1}·min^{-1}) are the rate constants of the pseudo-first-order and pseudo-second-order kinetic models, respectively, D_f: film diffusion coefficient; D_{ip}: intraparticle diffusion coefficient; a: material's average particle size.)

Kinetic Model		Equation	No.
Pseudo-first-order		$\frac{dq_t}{dt} = K_1(q_e - q_t)$	(Eq. S1)
Pseudo-second-order		$\frac{dq_t}{dt} = k_2(q_e - q_t)^2$	(Eq. S2)
Diffusion	Boundary layer diffusion	$\frac{q_t}{q_e} = 6\,(\frac{D_f}{\pi a^2})^{1/2}\sqrt{t}$	(Eq. S3)
	Intraparticle diffusion	$\mathrm{Ln}\left(1 - \frac{q_t}{q_e}\right) = \mathrm{Ln}\left(\frac{6}{\pi^2}\right) - \left(\frac{D_{ip}\,\pi^2}{a^2}\right) \mathrm{x}\ t$	(Eq. S4)

Table S2. Isotherm models used for the study of phenol recovery by SDGs-derived hydrochars (q_e (mg·g⁻¹) is the amount of adsorbate adsorbed at equilibrium, and C_e (mg·L⁻¹) represents the equilibrium concentration of the adsorbate in solution. For the Langmuir model, q_{max} (mg·g⁻¹) denotes the maximum monolayer adsorption capacity, and K_L (L·mg⁻¹) is the Langmuir constant related to the affinity between the adsorbent and the adsorbate. In the Freundlich model, K_F ((mg·g⁻¹)(L·mg⁻¹)$^{1/n}$) is the Freundlich constant indicating the adsorption capacity, nf represents the adsorption intensity or surface heterogeneity. Finally, in the Dubinin–Radushkevich model, q_m(mg·g⁻¹) is the theoretical adsorption capacity, K_{DR}(mol²·kJ⁻²) is the constant associated with the mean free energy of adsorption, and ε is the Polanyi potential, calculated using the equation $\varepsilon = RTln(1 + \frac{1}{C_e})$ where R is the universal gas constant (8.314 J·mol⁻¹·K⁻¹) and T is the absolute temperature (K).

**Isotherm Model**	**Equation**	**No.**
Langmuir	$q_e = q_{max} * \frac{K_L.Ce}{1+K_l.Ce}$	(Eq. S5)
Freundlich	$q_e = K_F\, Ce^{\frac{1}{n}}$	(Eq. S6)
Dubinin-Radushkevich	$q_e = q_m \exp(-K\varepsilon^2)$	(Eq. S7)

Table S3. Equations used for the calculus of phenol adsorbed and desorbed masses in column mode ($C_{Phen,0}$ and $C_{tr,0}$ represent the initial concentrations of phenol and the tracer respectively, $C_{Phen,V}$ and $C_{tr,V}$ denote the corresponding outlet concentrations of phenol and tracer measured after a given pumped volume V; V_{tot} is the total volume of water gathered at the column's exit at the end of the experiment, $C_{tr,v,i}$ and $C_{tr,v,i+1}$ are the concentrations of the tracer after gathered volumes at the exit of the column of 'V_i' and 'V_{i+1}', respectively; $C_{Phen,v,j}$ and $C_{Phen,v,j+1}$ are the phenol measured concentrations at times 'j' and 'j+1', which corresponded to water volume collected at the outlet of the column system of 'V_j' and 'V_{j+1}', respectively, $C_{des,Phen,v,k}$ and $C_{des,Phen,v,k+1}$ are the phenol measured desorbed concentrations at times 'k' and'k+1', corresponding to hot water volume collected at the column system's outlet of 'V_k' and 'V_{k+1}', respectively).

Phase	Equation	No.
Adsorption	$q_{col,ads} = \frac{M_{ads,Phen}}{M_{AHC}} = \frac{1}{M_{AHC}} * C_{0,Phen}\left[\int_0^{V_{tot}} \frac{C_{tr,v}}{C_{tr,0}} dv - \int_0^{V_{tot}} \frac{C_{Phen,v}}{C_{0,Phen}} dv\right]$	(Eq. S8)
	$= \frac{C_{0,Phen}}{2\, M_{AHC}}\left[\sum_{i=0}^{i=n}\left(\frac{C_{tr,v,i} + C_{tr,v,i+1}}{C_{tr,0}}\right)\left(V_{i+1} - V_i\right)\right] - \left[\sum_{j=0}^{j=n}\left(\frac{C_{Phen,v,j} + C_{Phen,v,j+1}}{C_{0,Phen}}\right)\left(V_{j+1} - V_j\right)\right]$	(Eq. S9)
Desorption	$q_{col,des} = \frac{M_{des,Phen}}{M_{AHC}} = \frac{\int_0^{V_{t,des}} C_{des,Phen}\, dv}{M_{AHC}}$	(Eq. S10)
	$q_{col,des} = \frac{1}{M_{AHC}}\left[\sum_{k=0}^{k=n}\left(\frac{C_{des,Phen,v,k} + C_{des,Phen,v,k+1}}{2}\right)\left(V_{k+1} - V_k\right)\right]$	(Eq. S11)

Table S4. Models used for the fitting of phenol BTCs in column mode

Model	equation	Parameters definition	No.
Thomas	$\frac{C}{C_0} = \frac{1}{1 + \exp(K_{TH}(\frac{q_{TH}M}{Q} - C_0 t)}$	C: Effluent concentration of the adsorbate at time t (mg L^{-1}); C_0: Influent concentration of the adsorbate (mg·L-1); K_{TH}: Thomas rate constant (mL min^{-1} mg^{-1}); q_{TH}: Maximum adsorption capacity (mg g^{-1}); M: Amount of adsorbent in the column (mg); Q: Volumetric flow rate (mL min^{-1}); t : time (h)	(Eq. S12)
Yoon–Nelson	$\frac{C}{C_0} = \frac{1}{1 + exp(K_{YN}(t - \tau))}$	K_{YN}: Yoon-Nelson rate constant (min-1); τ: time (min) required for 50% adsorbate breakthrough (h); τ corresponding to C/C0 = 0.5.	(Eq. S13)
Adams–Bohart	$\frac{C}{C_0} = exp(K_{AB} C_0 t - K_{AB} N_0 \frac{Z}{U})$	k_{AB} stands for the kinetic constant (L mg^{-1} min^{-1}); N_0 presents the concentration of saturation (mg L^{-1}); Z represents the column bed depth (cm); V is the linear velocity, which is determined as (flow rate/column's section area (cm min^{-1}).	Eq. (S14)

Table S5. Model parameters used for the fitting of the phenol experimental BTCs in column mode.

Model	Parameter	Assay for Q= 14 mL min^{-1}	Assay for Q= 26 mL min^{-1}
Bohart-Adams	K_{AB} (L mg^{-1} min^{-1})	0.0014	0.0015
	N_0 (mg L^{-1})	7725	7820
	R^2	0.861	0.606
	$MAPE_{col}$ (%)	64.0	95.1
Thomas	K_{Th} (L mg^{-1} min^{-1})	0.0022	0.0036
	q_{TH} (mg/g)	43.2	37.1
	q_{exp}	42.3	36.7
	R^2	0.989	0.989
	$MAPE_{col}$ (%)	16.5	15.1
Yoon-Nelson	K_{YN} (min-1)	0.0170	0.0317
	τ_{calc} (min)	788.7	327.2
	τ_{exp} (min)	779.7	316.6
	R^2	0.989	0.989
	$MAPE_{col}$ (%)	16.5	15.1
